# RBM3-associated germline variants and their functional role in gastric cancer susceptibility and progression

**DOI:** 10.3389/fonc.2026.1790197

**Published:** 2026-04-01

**Authors:** Qingsheng Zheng, Shuai Peng, Xueying Wu

**Affiliations:** Department of General surgery, The Affiliated People’s Hospital of Fujian University of Traditional Chinese Medicine, Fuzhou, Fujian, China

**Keywords:** apoptosis, bioinformatics analysis, gastric cancer, public databaseresearch, RBM3, single nucleotide polymorphism

## Abstract

**Background:**

Gastric cancer (GC) is the fifth most commonly diagnosed malignancy and the fourth leading cause of cancer-related mortality worldwide. RNA-binding motif protein 3 (RBM3) has been associated as a prognostic marker in several cancers; however, its genetic contribution and functional role in gastric cancer remain unclear.

**Methods:**

Genome-wide association study (GWAS) data was integrated with experimental validation to investigate the role of RBM3 in GC. RBM3-associated expression quantitative trait loci (eQTLs) were identified from a GWAS of 3,301 individuals and evaluated for their association with GC risk using a large-scale GWAS comprising 456,348 individuals. Colocalization analysis was performed using stomach tissue eQTL data. RBM3 expression was assessed in 60 paired GC and adjacent normal tissues and in multiple GC cell lines. Functional effects of RBM3 modulation were examined through proliferation, colony formation, migration, invasion, and xenograft assays.

**Results:**

Several independent variants within the RBM3 locus showed a consistent protective association with GC risk and were linked to RBM3 expression regulation. RBM3 expression was significantly reduced in GC tissues compared with adjacent normal tissues (P < 0.001). Low RBM3 expression correlated with advanced tumor stage (III–IV), lymph node and distant metastasis, and larger tumor size. Functionally, RBM3 overexpression inhibited GC cell proliferation, clonogenicity, migration, and invasion *in vitro*. *In vivo*, RBM3 overexpression suppressed xenograft tumor growth, reduced Ki67-positive proliferation, and enhanced apoptotic activity.

**Conclusion:**

Our findings demonstrate that RBM3 acts as a genetically supported tumor suppressor in gastric cancer. RBM3-associated germline variants contribute to GC susceptibility, and RBM3 downregulation promotes aggressive tumor behavior. RBM3 therefore represents a promising biomarker and potential therapeutic target in gastric cancer.

## 1 Introduction

Gastric cancer (GC) is ranked as fifth most commonly diagnosed malignancy and thus remains as major global health burden. It is the fourth top cause of cancer-related death throughout the globe (1). In 2020 alone, GC was responsible for approximately 1.09 million new cases and 0.77 million deaths (2). In spite of gradual decrease in incidence in some regions, the outcomes for gastric cancer are often poor. This is poor mainly due to late diagnosis and hostile course of disease. Most patients are presented with advanced stage of the disease. It results into high rates of recurrence and metastasis after treatment. Likewise it also contributes to low rates of survival. Indeed, an important challenge in management of gastric cancer is lack of early detection methods (3). The conventional tumor biomarkers such as CEA, CA19-9, and CA72–4 for the diagnosis of cancer have very low sensitivity and poor specificity. This limitation highlights the need for new biomarkers and therapeutic targets. This novelty is required for improvement in early diagnosis and outcomes of patient in Gastric Cancer (4).

The etiology of gastric cancer (GC) is multifactorial. It involves a complex interaction of environmental, lifestyle, and genetic factors. Some risk factors include chronic *Helicobacter pylori* infection, salty diets or smoked foods, tobacco smoke, and low socioeconomic status. However, these exposures alone do not fully account for substantial geographic and inter-individual variability observed in risk of Gastric Cancer (5). The additional and important component in pathogenesis of gastric cancer is inherited genetic susceptibility. Notably, family history of gastric cancer is associated with an increased risk. Also, rare hereditary syndromes demonstrate profound impact that germline genetic variation can have on development of GC (6). Beyond these mutations, accumulation of evidence from genome-wide association studies (GWAS) indicates that more common germline variants also reduce risk of GC (7). Over past decade, GWAS conducted largely in East Asian populations have identified multiple susceptibility loci. It had shown that genes such as *MUC1*, *PSCA*, *PRKAA1*, *ZBTB20*, and others are involved in genetic predisposition to GC (8). Together, these findings highlight germline genetics as an emergent frontier in to understand GC etiology and complements established environmental drivers. Nevertheless, most identified variants confer only modest risk increments and collectively explain limited proportion of heritable susceptibility. A key challenge remains to clarify the biological mechanisms through which these genetic factors contribute to gastric tumorigenesis and to identify the causal genes underlying these loci that may serve as biomarkers or therapeutic targets (9).

In this context, RNA-binding motif protein 3 (*RBM3*) has gained attention as potential tumor suppressor and biomarker in cancer (10). *RBM3* encodes small cold-inducible RNA-binding protein that is evolutionarily conserved and broadly expressed. Under conditions of mild hypothermia or other cellular stresses, RBM3 expression is strongly upregulated and suggests to have a role in the stress response. Functionally, RBM3 is known to bind transcripts with AU-rich elements and promote global protein synthesis. It stabilizes specific mRNAs and enhances microRNA processing (11). Through these activities, RBM3 generally exerts pro-survival and cytoprotective effects in cells. For instance, RBM3 induction has been shown to protect neurons and muscle cells from hypoxic or toxic insults by prevention of apoptosis and preservation of cellular integrity. Such observations position RBM3 as a pleiotropic regulator of cell fate under stress, with potential relevance to cancer development and progression (12).

Intriguingly, RBM3 appears to have dual roles in cancer biology. On one hand, early experimental studies indicated that RBM3 can function in a pro-tumorigenic manner and helps in survival and proliferation of cancer cells (13). In colorectal cancer models, RBM3 was reported to act as a proto-oncogene that enhances translation of growth- and angiogenesis-related mRNAs such as *COX-2*, *IL-8*, and *VEGF* (11).The RBM3 overexpression in colon epithelial cells increased their proliferation and anchorage-independent growth, whereas RBM3 knockdown led to reduced *COX-2* production, cell-cycle arrest, and apoptosis. These findings suggest that in the stressful tumor microenvironment, RBM3 can promote tumor cell survival pathways that favor cancer growth (13). On the other hand, a growing body of clinical evidence correlates RBM3 with *favorable* cancer outcomes, implying a tumor-suppressive influence *in vivo*. Numerous immunohistochemical studies across cancer types have found RBM3 to be upregulated in tumors relative to normal tissues, yet high RBM3 expression within tumors is paradoxically associated with *better* patient prognosis (14). The elevated level of RBM3 correlate with less aggressive disease features and longer patient survival. This creates RBM3 as hopeful predictive biomarker. This pattern is particularly obvious in gastrointestinal cancers. In adenocarcinomas of esophagus and stomach, the tumors which exhibit high nuclear RBM3 expression are more frequently of less aggressive intestinal subtype. These show significantly reduced risks of recurrence and death as compared to RBM3-low tumors. These findings are consistent with the study by Jonsson et al., who reported that high RBM3 expression in esophageal and gastric adenocarcinoma was associated with intestinal metaplasia-related tumors and independently predicted reduced recurrence and improved survival (15). Similarly, in colorectal cancer, loss of RBM3 is linked to advanced stage and poor outcome. On the otherhand, strong RBM3 expression predicts improvement of survival (16). A study in patients of gastric cancer found that those with RBM3-high tumors had markedly longer overall survival. In multivariate analysis, high RBM3 was an independent predictor of reduced mortality risk. Together, these data suggest that RBM3 may act to restrain tumor progression and can behave as tumor suppressor (17).

Despite evidence which suggest that RBM3 restrains tumor aggressiveness, there are certain critical gaps which play its role in gastric cancer. The contribution of germline RBM3 variation to risk of GC is unknown. Although expression of RBM3 correlates with prognosis, its functional role and mechanisms in gastric tumorigenesis have not been established. These gaps are essential to be address to clarify GC biology and reveal RBM3 as a potential biomarker or therapeutic target. To address these gaps, the present study adopted integrative approach that merges population-level genetic analysis with experimental biological validation. Our study used GWAS data to examine whether germline single nucleotide polymorphisms (SNPs) at RBM3 locus show consistent links with risk of GC. This study then conducted widespread *in-vivo* and *in-vitro* studies for determination of biological effects of RBM3 modulation in GC models. By combination of genetic association evidence with mechanistic assays, this study aims to move beyond statistical correlation. It also helps to directly establish the role of RBM3 in gastric carcinogenesis. The findings of this study provide new insight into how RBM3-linked germline variation may contribute to interindividual differences in GC susceptibility and highlight RBM3’s functional impact as a tumor suppressor in the context of gastric cancer.

## 2 Materials and methods

### 2.1 Data retrieval

Using *RBM3* as a search term in the GWAS Catalog (https://www.ebi.ac.uk/gwas/), we identified an expression quantitative trait loci (eQTL) GWAS (GCST90242696) assessing genetic variants associated with *RBM3* expression in 3,301 individuals of European ancestry. 8511609 SNP data existed in GCST90242696 (**Supplementary Table 1**). The relatively modest sample size reflects the molecular phenotype analyzed rather than a disease-based GWAS. Then using gastric cancer as a search term, we found the GWAS summary-level data with registration ID GCST90041806. Whole genome sequencing data of 456348 European samples were included in GCST90041806, and 11796992 SNP data existed in GCST90041806 (**Supplementary Table 1**). In this study, GCST90242696 was used to identify RBM3-associated genetic variants, and GCST90041806 was used to evaluate their associations with gastric cancer risk.

### 2.2 Genetic association analysis

GWAS summary-level data for RBM3-associated variants and gastric cancer were downloaded from public repositories. Single nucleotide polymorphisms (SNPs) located within or near the *RBM3* locus were selected using minor allele frequency (MAF) > 0.01, imputation INFO score > 0.8, and linkage disequilibrium pruning at r² < 0.1 based on the European 1000 Genomes reference panel. Variants with P < 1 × 10^-5^ were considered suggestively associated. Variants showing suggestive association with RBM3 expression or reported eQTL effects were prioritized. Association effect estimates were examined to identify RBM3-linked variants exhibiting a directionally consistent association with reduced gastric cancer risk. Colocalization analysis was performed using the COLOC framework with stomach tissue eQTL data from GTEx v8 to evaluate shared regulatory signals.

### 2.3 Patient samples and tissue collection

Briefly, 60 GC tissues and along with their paired adjacent non-tumorous tissues were collected. Prior to sample collection, each patient provided written informed consent. This study was carried out in accordance with the Declaration of Helsinki and the institutional research committee’s ethical guidelines. The Fujian University of Traditional Chinese Medicine’s Affiliated People’s Hospital Ethics Committee authorized the collection of clinical samples (Approval No. 2023-002-02). Before analysis, all tumor tissues were immediately frozen in liquid nitrogen and stored at −80 °C. Total RNA was extracted from gastric cancer tissues and matched adjacent normal tissues for quantitative real-time PCR (qPCR) analysis of RBM3 expression. In addition, a subset of frozen tissue samples was used for protein extraction and subsequent Western blot analysis to evaluate RBM3 protein expression. Detailed procedures for Western blot analysis are described in Section 2.5. RBM3 mRNA expression levels in gastric cancer tissues were subsequently classified into high- and low-expression groups based on the median RBM3 expression value among the 60 tumor samples for clinicopathological correlation analysis.

### 2.4 Cell culture and transfection

The normal gastric epithelial cell line GES-1 and human gastric cancer cell lines (HGC27, AGS, MKN28, MKN45, SNU601, and SNU668) were obtained from the Cell Bank of the Chinese Academy of Sciences and authenticated by the supplier. Among the gastric cancer cell lines examined, AGS and HGC-27 cells were selected for subsequent functional experiments because they exhibited relatively lower endogenous RBM3 expression and represent distinct biological characteristics of gastric cancer. All *in vitro* functional assays were independently performed in both AGS and HGC-27 cells to ensure reproducibility across two distinct gastric cancer cell models. To account for potential heterogeneity in RBM3 expression and function in gastric cancer, functional experiments were performed using two biologically distinct gastric cancer cell lines (AGS and HGC-27). RBM3 overexpression experiments were conducted in AGS cells, whereas RBM3 knockdown experiments were performed in HGC-27 cells. This complementary gain- and loss-of-function strategy enabled evaluation of RBM3 effects across distinct gastric cancer cellular contexts. These two cell lines are also widely used and well-characterized models for investigating gastric cancer proliferation, migration, invasion, and tumorigenicity. Dulbecco’s Modified Eagle Medium (DMEM) was supplemented with 10% foetal bovine serum (FBS) and 1% penicillin-streptomycin. This media was used to cultivate the cells. All cells were cultured with 5% CO_2_ at 37 °C in the incubator. For transfection, cells were either stably transfected with RBM3 shRNA constructs or transiently transfected with RBM3 overexpression plasmids by Lipofectamine 3000 (Invitrogen, USA) based on the standard protocol. RBM3 modulation efficiency was primarily evaluated at the transcript level using quantitative real-time PCR (qPCR), which is widely used to assess gene expression changes following shRNA-mediated knockdown or plasmid-driven overexpression. Two independent short-hairpin RNA constructs targeting different regions of RBM3 mRNA (designated shRBM3–1 and shRBM3-2) were used to achieve stable RBM3 knockdown and to minimize off-target effects. A non-targeting shRNA was used as the negative control (sh-NC). Puromycin (2 μg/mL) were added to generated stable transfectants cells. Stable RBM3 knockdown cells were used for colony formation assays, whereas RBM3 overexpression cells were used for xenograft experiments.

### 2.5 Western blot analysis

Protein expression was assessed by Western blot. Total protein was extracted from clinical tissue samples or cultured cell lines using RIPA lysis buffer (Beyotime, Shanghai, China) supplemented with 1% phenylmethylsulfonyl fluoride (PMSF; Beyotime). Protein concentrations were determined using a bicinchoninic acid (BCA) protein assay kit (Beyotime) according to the manufacturer’s instructions. Equal amounts of protein (20–30 μg) were separated by 10–12% SDS–polyacrylamide gel electrophoresis (SDS–PAGE) and transferred onto polyvinylidene fluoride (PVDF) membranes (Millipore, USA). Membranes were blocked with 5% skimmed milk for 1 h at room temperature and incubated overnight at 4 °C with primary antibodies against RBM3 (ab134946, Abcam, Cambridge, UK; 1:1000 dilution) and β-actin (Affinity Biosciences, Cincinnati, OH, USA; 1:5000 dilution) as the loading control. After washing with TBST, membranes were incubated with horseradish peroxidase (HRP)-conjugated secondary antibodies for 1 h at room temperature. Protein bands were visualized using an enhanced chemiluminescence (ECL) detection kit (SeraCare, USA) and imaged with a ChemiDoc Touch Imaging System (Bio-Rad, USA). Densitometric analysis of band intensities was performed using Image Lab software (Bio-Rad). Detailed antibody information is provided in **Supplementary Table 5**.

### 2.6 CCK-8 proliferation assay

Cell Counting Kit-8 (CCK-8; Beyotime, China) was used to measure cell proliferation. After seeding 5000 cells onto 96-well plates, CCK-8 reagent was added 24, 48, and 72 hours later, and the plates were incubated for one to two hours at 37 °C. In order to compare the growth rates of the groups of normal and transfected cells, proliferation curves were created and measured at 450 nm. For proliferation assays, Non-targeting shRNA-transfected cells (sh-NC) and empty vector-transfected cells were used as standard experimental controls for knockdown and overexpression experiments, respectively. These control groups account for nonspecific effects associated with transfection procedures and serve as baseline references for evaluating RBM3-specific effects on cell proliferation.

### 2.7 Colony formation assay

To enable colony development, 500 cells were planted into 6-well plates and cultured for 10–14 days under normal conditions. Crystal violet was used to dye the cells after they had been fixed with 4% paraformaldehyde. After crystal violet staining, colonies were counted across the entire well rather than selected microscopic fields. Colony numbers were normalized to the respective control group (sh-NC for knockdown experiments and vector for overexpression experiments), which was defined as 1.0, and presented as relative colony numbers. Representative images show whole-well views, and colony numbers were quantified based on predefined counting criteria and averaged from three independent experiments.

### 2.8 Migration and invasion assays

Cell migration and invasion were assessed using the Transwell test with chambers (Corning, USA). Transfected cells were seeded in serum-free media into the upper chambers for migration tests, and 10% foetal bovine serum was introduced to the lower chambers as a chemoattractant. The upper chambers were precoated with Matrigel to simulate the extracellular matrix for invasion tests. Cells that had migrated or invaded were fixed, stained, and evaluated by counting cells in five randomly chosen microscopic areas per membrane following a 24-hour incubation period at 37 °C. Representative images were captured under identical imaging conditions, and scale bars indicate 100 µm.

### 2.9 *In vivo* tumorigenesis assay

The tumor-suppressive effects of RBM3 were further assessed using a subcutaneous xenograft nude mice model. 6-week-old mice were divided randomly into two groups (n=6 each group): vector-transfected cells and RBM3-overexpressing cells. Mice were subcutaneously injected in the flanks with 5 × 10^6^ AGS cells, and tumor volume were measured twice a week with calipers. Tumor volume was calculated using the formula: (length × width²)/2. Four weeks later, mice were anesthetized with 4% isoflurane and euthanized by cervical dislocation under deep anesthesia. Then tumors were excised, weighed, and processed for histopathological analysis. All animal studies were carried out in accordance with the institutional guidelines for animal care and use, and were approved by the Ethics Committee of the Affiliated People’s Hospital of Fujian University of Traditional Chinese Medicine (Approval No. 084-2).

### 2.10 Histological and immunohistochemical analysis

Tumour tissues were embedded in paraffin. It was then stained with haematoxylin and eosin (H&E) after being fixed in 10% formalin. Immunohistochemistry was performed using primary antibodies against RBM3 (ab134946, Abcam, Cambridge, UK; 1:300 dilution) and Ki-67 (#AF0198, Affinity Biosciences, Cincinnati, OH, USA; 1:200 dilution) to evaluate RBM3 expression and tumor cell proliferation. After incubation with appropriate secondary antibodies, signals were visualized using DAB substrate and counterstained with hematoxylin. Detailed antibody information is provided in **Supplementary Table 5**. Apoptosis was assessed using a TUNEL assay following the manufacturer’s protocol. All histological and immunohistochemical images were acquired under identical imaging settings, and scale bars indicate 50 µm.

### 2.11 Statistical analysis

R (version 4.2.0) and GraphPad Prism (version 9.0) were used to perform all statistical analyses. Mean ± standard deviation (SD) was used to express results from animal and cell-based investigations. The Student’s t-test was used to compare two groups, and Tukey’s *post-hoc* test and one-way analysis of variance (ANOVA) were used to analyze multiple-group comparisons. The chi-square test or, when suitable, Fisher’s exact test were used to compare categorical variables in clinical tissue analysis.

For the genetic component of this study, summary-level single nucleotide polymorphism (SNP) data for RBM3 and gastric cancer were extracted from publicly available genome-wide association studies (GWAS). SNPs were filtered according to imputation quality, minor allele frequency, and linkage disequilibrium constraints to ensure independence. Effect estimates for SNP–disease associations were summarized descriptively, and variants demonstrating consistent protective directions were highlighted. No causal inference modelling was performed. Spearman’s rank correlation coefficients were used to analyze the relationship between RBM3 expression level and clinicopathological parameters. Two-way ANOVA was applied to analyze tumor volume data in xenograft experiments. All statistical tests were two-sided, and statistical significance was set at P < 0.05.

## 3 Results

### 3.1 Identification of RBM3-related genetic variants

**Figure 1:**
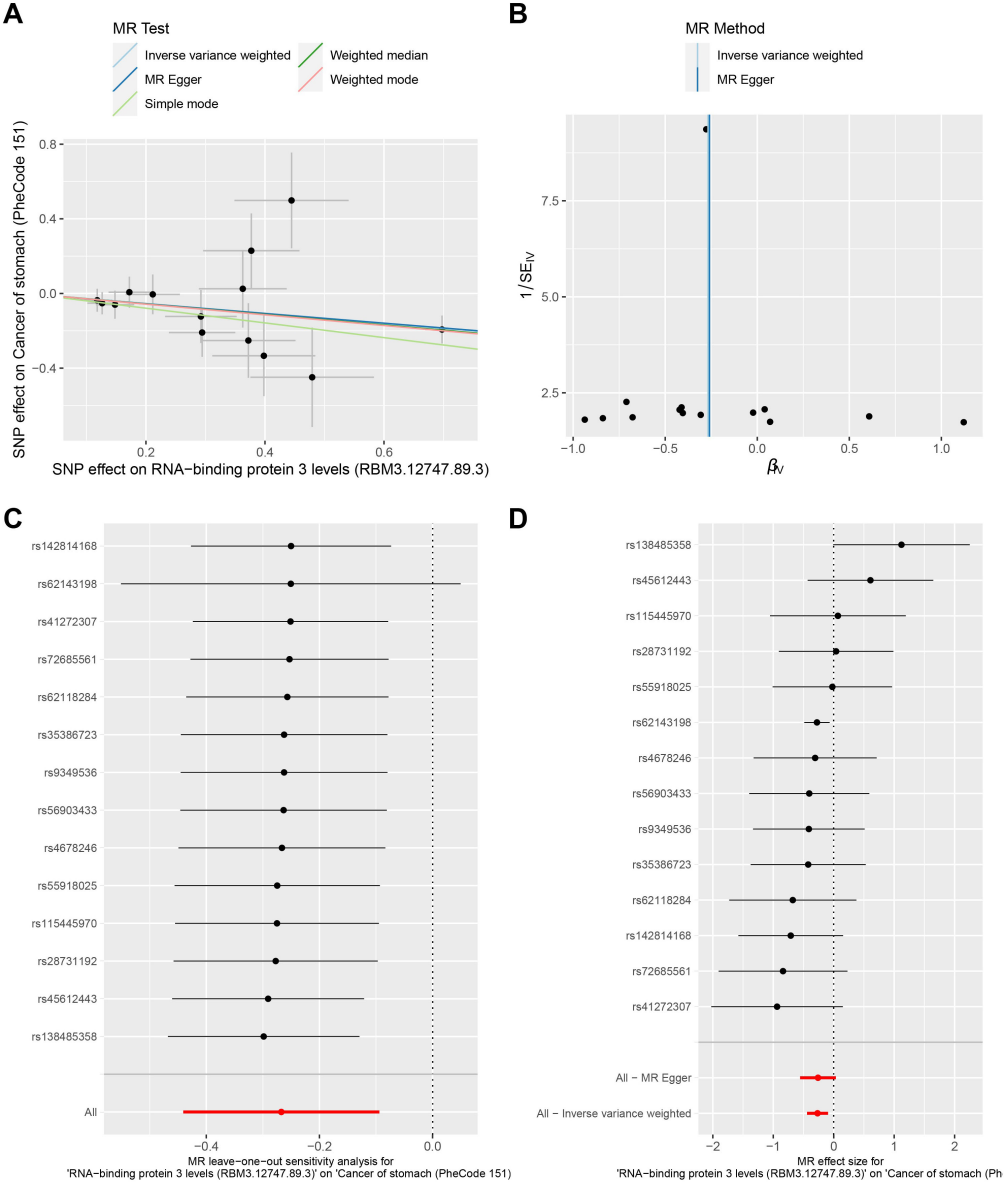
Genetic association patterns of RBM3-related variants with gastric cancer risk. **(A, B)** Scatter plots showing the association effect estimates of RBM3-associated single nucleotide polymorphisms (SNPs) with gastric cancer risk based on GWAS summary statistics. Each point represents an independent SNP within or near the *RBM3* locus. **(C, D)** Sensitivity analyses illustrating the consistency of association patterns after sequential exclusion of individual SNPs, indicating that no single variant disproportionately drives the observed signal.

Analysis of available GWAS summary data identified multiple common variants located within or near the *RBM3* locus that exhibited a directionally consistent association with reduced gastric cancer risk. Although individual variants did not reach genome-wide significance, the concordant association pattern across independent SNPs suggests a potential contribution of inherited variation in the *RBM3* region to inter-individual variability in gastric cancer risk (**Figures 1A–D**; **Supplementary Table 2**). Furthermore, interrogation of public eQTL resources indicated that a subset of these variants was associated with RBM3 expression levels, supporting a potential regulatory link between germline variation and RBM3 transcriptional activity, which provides a biological basis for the observed genetic association with gastric cancer susceptibility. These genetic observations provided a population-level rationale for subsequent functional investigation of RBM3 in gastric cancer (**Supplementary Tables 3**, **4**).

### 3.2 RBM3 expression level

**Figure 2:**
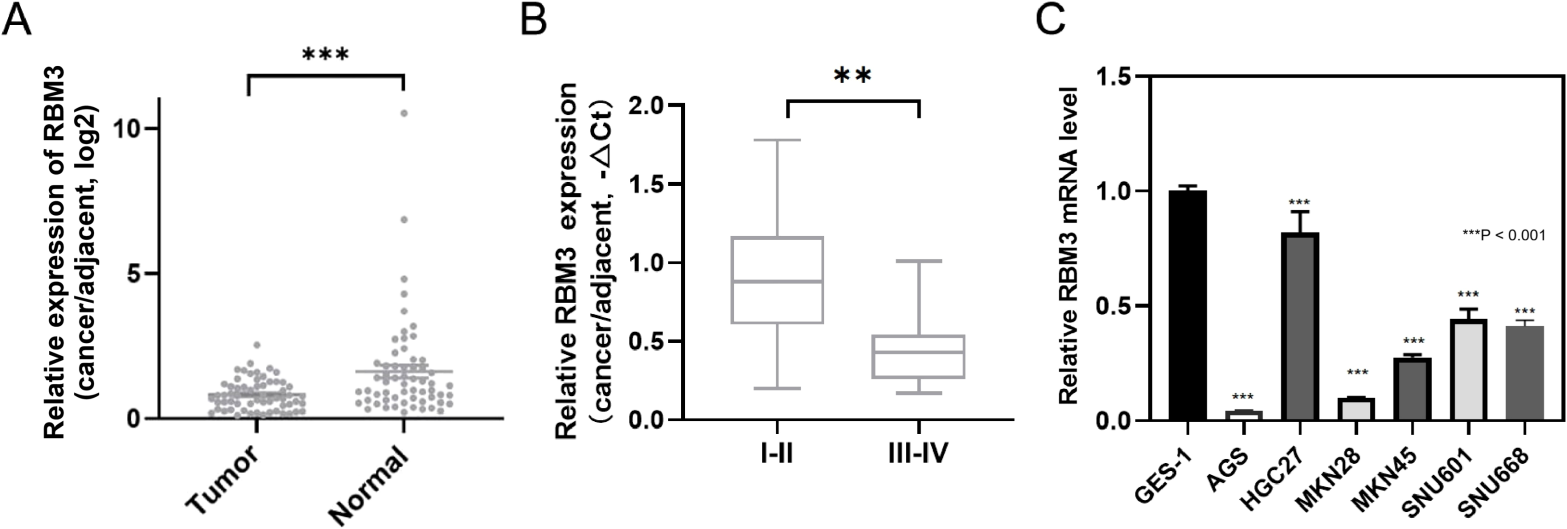
Downregulation of RBM3 expression in gastric cancer tissues and cell lines. **(A)** Quantitative real-time PCR (qPCR) analysis showing RBM3 expression levels in gastric cancer tissues compared with paired adjacent normal gastric epithelial tissues. RBM3 mRNA expression levels were measured by qPCR in 60 paired gastric cancer tissues and adjacent non-tumorous tissues (n = 60 per group). **(B)** Association between RBM3 expression levels and clinical stage of gastric cancer. **(C)** qPCR analysis of RBM3 expression in normal gastric epithelial cells and multiple gastric cancer cell lines. Data are presented as mean ± SD from three independent experiments. Statistical significance was analyzed using Student’s t-test or one-way ANOVA followed by Tukey’s *post hoc* test, as appropriate. In panel **(C)**, ***P < 0.001 compared with the normal gastric epithelial cell line (GES-1) using one-way ANOVA followed by Tukey’s *post hoc* test. **P < 0.01.

Quantitative real-time PCR (qPCR) analysis revealed significantly lower levels of *RBM3* expression in gastric cancer tissues compared with paired neighboring normal gastric epithelial tissues (**Figure 2A**). This differential expression showed and suggested that RBM3 may play role in progression of gastric cancer. Further clinicopathological correlation analysis was performed to evaluate the clinical significance of RBM3 expression in gastric cancer patients (**Table 1**). RBM3 expression levels were dichotomized into high and low groups based on the median expression value. As shown in **Table 1**, low RBM3 expression was significantly associated with aggressive clinicopathological features. Specifically, patients with larger tumor size (>5 cm) exhibited significantly lower RBM3 expression compared with those with smaller tumors (P = 0.004). In addition, reduced RBM3 expression was strongly correlated with advanced pathological T stage (T3–T4 vs. T1–T2, P = 0.0009), lymph node metastasis (N1–2 vs. N0, P < 0.0001), and the presence of distant metastasis (M1 vs. M0, P = 0.0107). Moreover, low RBM3 expression was significantly enriched in patients with poorly differentiated tumors (P = 0.0438) and advanced tumor stage (stage III–IV vs. I–II, P = 0.0039). No statistically significant associations were observed between RBM3 expression and patient age or gender. Collectively, these results indicate that decreased RBM3 expression is closely associated with tumor aggressiveness and disease progression in gastric cancer, which is consistent with previous clinical studies reporting that RBM3 downregulation correlates with advanced stage and metastatic potential in gastric cancer. It indicated that downregulation RBM3 correlates with more aggressive clinicopathological features (**Figure 2B**; **Table 1**).

**Table 1:** Association between RBM3 expression levels and clinicopathological characteristics in 60 gastric cancer patients.

Variable		N	RBM3 expression (low)	RBM3 expression (high)	P value
All cases		60	30	30	—
Age (years)	≥65	38	22	16	0.0645
	<65	22	7	15	
Gender	Male	33	17	16	0.793
	Female	27	15	12	
Tumor size (cm)	≤5	32	10	22	0.004
	>5	28	20	8	
Pathological T stage	T1–T2	38	10	28	0.0009
	T3–T4	22	16	6	
Lymph node status	N0	40	10	30	<0.0001
	N1–2	20	16	4	
Distant metastasis status	M0	32	12	20	0.0107
	M1	28	20	8	
Histologic grade	Well	19	13	6	0.0438
	Moderate	34	13	20	
	Poor	7	4	4	
Tumor stage	I–II	36	10	26	0.0039
	III–IV	24	16	8	

RBM3 expression levels were dichotomized into low and high groups based on the median expression value. Statistical significance was defined as P < 0.05. Fisher’s exact test was applied when expected cell counts were <5.

### 3.3 Functional assays of RBM3 in GC cells

RBM3 expression levels were examined in normal gastric epithelial cells and multiple gastric cancer cell lines. Consistent with public transcriptomic resources, RBM3 expression exhibited heterogeneity across different gastric cancer cell lines, while overall RBM3 levels were reduced in cancer cell lines compared with normal gastric epithelial cells (**Figure 2C**). Minor discrepancies between public CCLE/HPA datasets and our qPCR measurements may reflect differences in cell line passage, culture conditions, and technical platforms.

**Figure 3:**
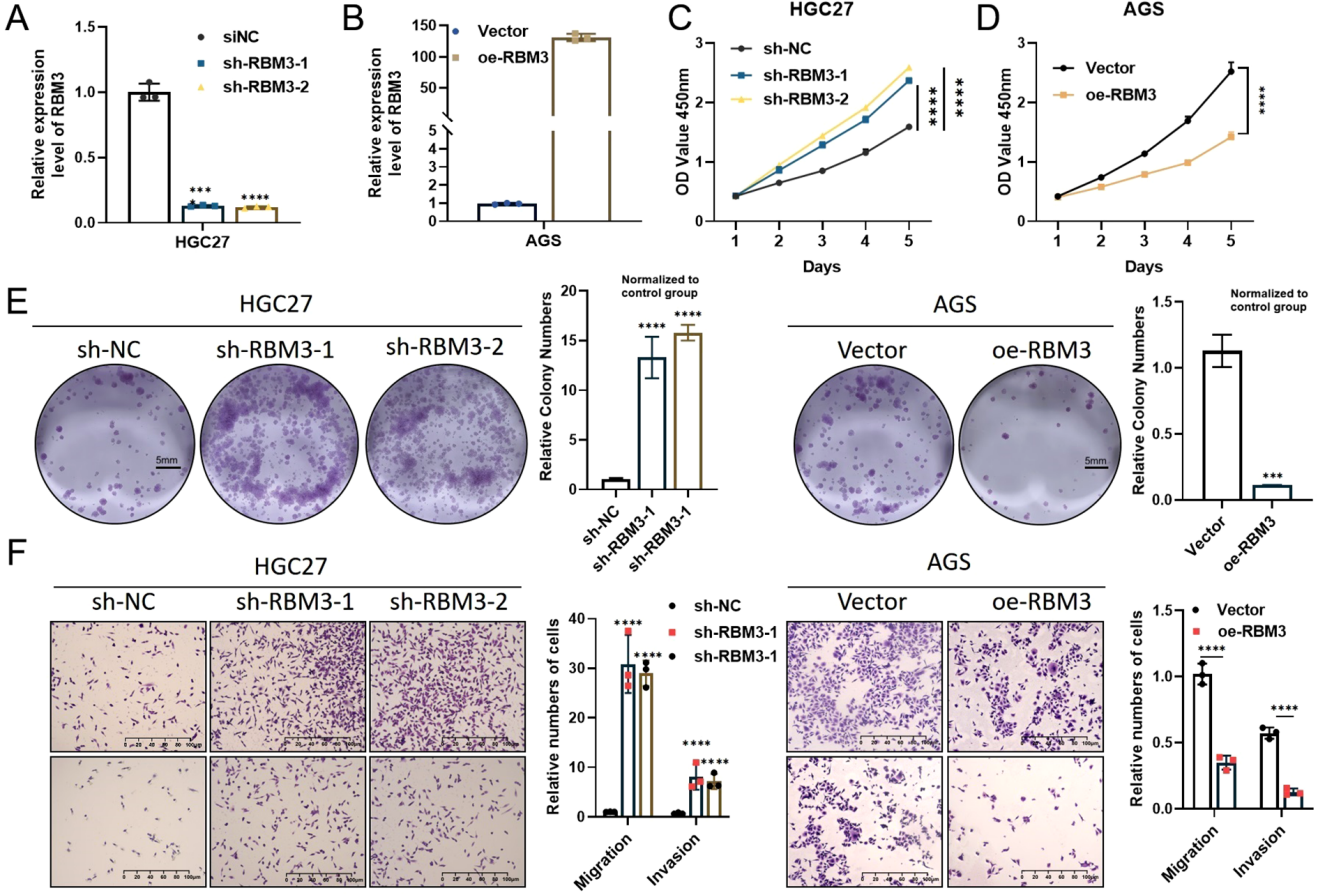
Effects of RBM3 modulation on gastric cancer cell proliferation, migration, and invasion *in vitro*. **(A)** qPCR analysis confirming RBM3 knockdown efficiency in HGC-27 cells transfected with shRBM3 constructs. **(B)** qPCR analysis confirming RBM3 overexpression efficiency in AGS cells following RBM3 plasmid transfection. **(C, D)** CCK-8 assays showing the effects of RBM3 overexpression and knockdown on the proliferation of HGC27 and AGS cells. sh-NC and vector-transfected cells served as the respective control groups for CCK-8 proliferation assays. **(E)** Colony formation assays evaluating the impact of RBM3 knockdown and overexpression on clonogenic capacity. Representative images show whole-well colony formation. Colony numbers were quantified across the entire well and presented as relative colony numbers normalized to the control group (sh-NC or vector), which was defined as 1.0. Scale bar = 5 mm. **(F)** Transwell migration and invasion assays demonstrating the effects of RBM3 overexpression and knockdown on the migratory and invasive abilities of gastric cancer cells. Representative Transwell migration and invasion images are shown. Scale bar = 100 µm. Data are presented as mean ± SD from three independent experiments. Student’s t-test was used for two-group comparisons, and one-way ANOVA followed by Tukey’s *post hoc* test was applied for multiple comparisons. sh-NC or vector-transfected cells served as control groups. ***P < 0.001, ****P < 0.0001.

**Figure 4:**
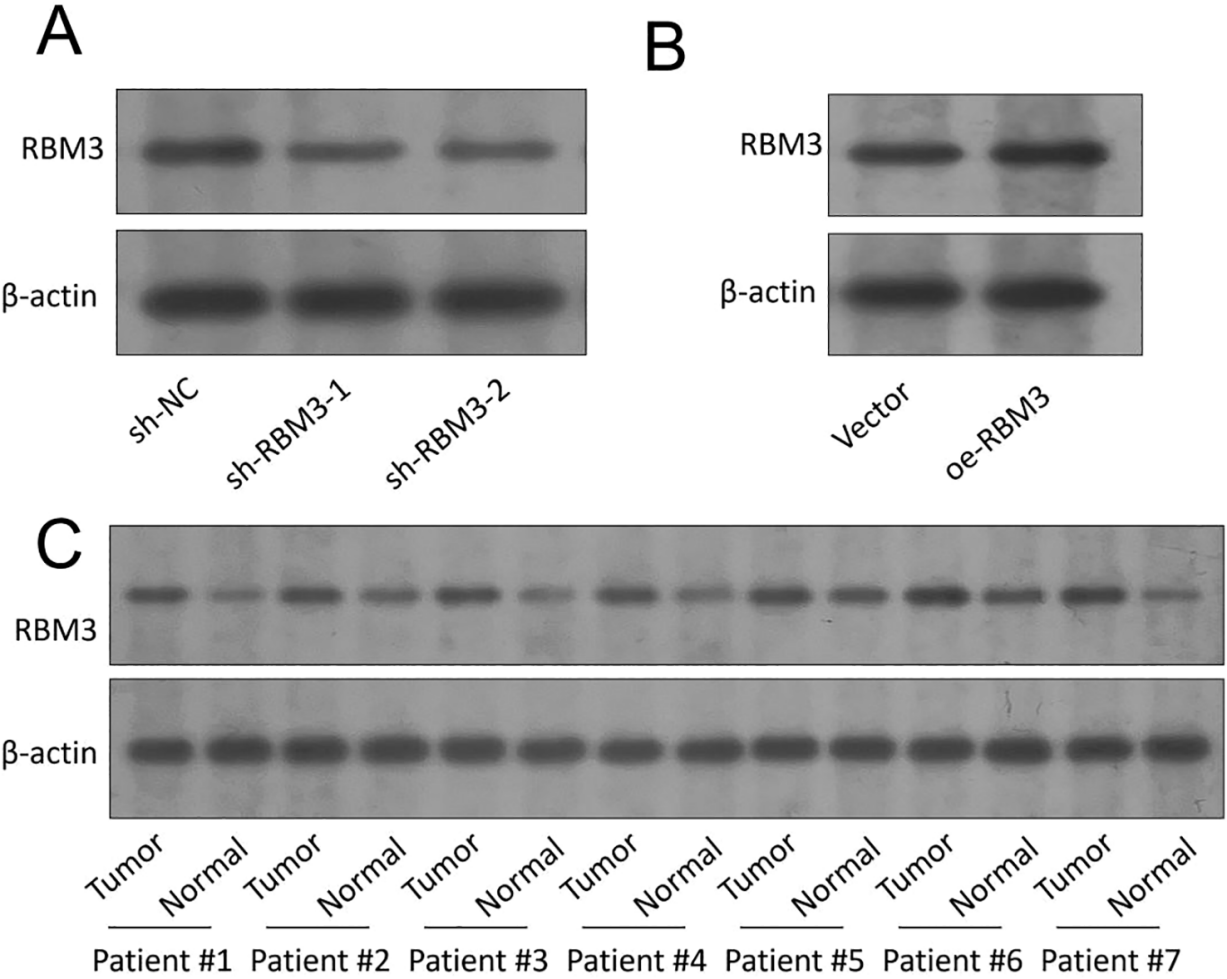
Validation of RBM3 protein expression in gastric cancer cell lines and clinical tissues. **(A)** Representative Western blot analysis showing RBM3 protein expression in HGC-27 cells transfected with non-targeting control (sh-NC) or RBM3-targeting shRNAs (shRBM3–1 and shRBM3-2). **(B)** Representative Western blot analysis showing RBM3 protein expression in AGS cells transfected with RBM3 overexpression plasmid (oe-RBM3) or empty vector control. **(C)** Representative Western blot analysis of RBM3 protein expression in seven paired gastric cancer (GC) tissues and matched adjacent non-cancerous tissues selected from the clinical cohort (n = 7 pairs). β-actin was used as the loading control.

The biological role of RBM3 in gastric cancer was further investigated using a series of functional assays conducted in AGS and HGC-27 cells, which were selected based on their relatively low basal RBM3 expression and their suitability as representative gastric cancer models. Firstly, quantitative real-time PCR (qPCR) was performed to verify RBM3 knockdown efficiency in HGC-27 cells transfected with shRBM3 constructs and RBM3 overexpression efficiency in AGS cells following plasmid transfection (**Figures 3A, B**). These analyses confirmed efficient RBM3 knockdown in HGC-27 cells and successful RBM3 overexpression in AGS cells at the mRNA level. To further validate RBM3 modulation at the protein level, Western blot analysis was performed. As shown in **Figure 4A**, RBM3 protein expression was significantly reduced in HGC-27 cells transfected with shRBM3 constructs compared with the sh-NC control. Conversely, RBM3 protein levels were markedly increased in AGS cells following RBM3 overexpression (**Figure 4B**). In addition, Western blot analysis of paired gastric cancer tissues and adjacent normal tissues demonstrated that RBM3 protein expression was generally lower in tumor tissues compared with matched normal tissues (**Figure 4C**). These results confirm successful RBM3 modulation and support the reduced RBM3 expression observed in gastric cancer. These findings confirm the successful modulation of RBM3 expression at both the transcript and protein levels. To minimize potential off-target effects, two independent RBM3-targeting shRNA constructs (shRBM3–1 and shRBM3-2) were used, both of which produced consistent knockdown efficiency and concordant functional phenotypes. CCK-8 proliferation assays showed that RBM3 knockdown significantly increased proliferative capacity, whereas RBM3 overexpression significantly reduced proliferative capacity compared with their respective control groups (sh-NC or vector) (**Figures 3C, D**). Similarly, Colony formation assays showed that RBM3 knockdown significantly increased relative colony formation ability, whereas RBM3 overexpression significantly reduced relative colony formation ability compared with the respective control groups (**Figure 3E**). Transwell migration and invasion assays further demonstrated that overexpression of RBM3 considerably suppressed migratory & invasive abilities of GC cells (**Figure 3F**).

### 3.4 *In vivo* tumorigenesis

**Figure 5:**
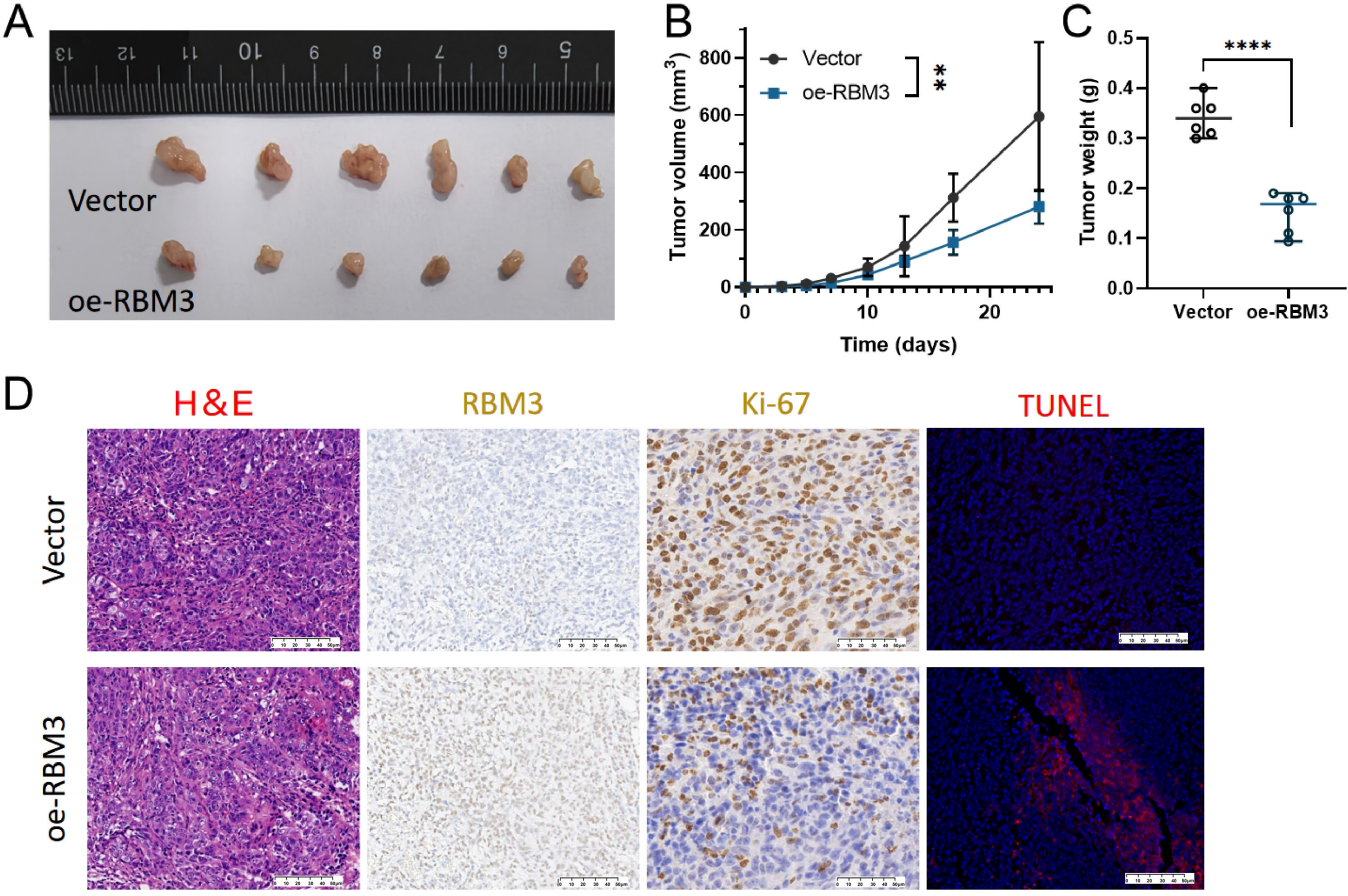
Effects of RBM3 overexpression on gastric cancer growth *in vivo*. **(A)** Schematic overview of the subcutaneous xenograft mouse model using AGS cells with RBM3 overexpression or vector control. **(B, C)** Tumor volume growth curves and final tumor weights in control and RBM3-overexpressing groups. **(D)** Representative images of hematoxylin and eosin (H&E) staining, Ki67 immunohistochemistry, RBM3 immunohistochemistry, and TUNEL staining of tumor sections from control and RBM3-overexpressing xenografts. Representative images of H&E staining, RBM3 immunohistochemistry, Ki-67 immunohistochemistry, and TUNEL staining in xenograft tumors. Images were acquired using a 20× objective lens; scale bar = 50 μm. Data are presented as mean ± SD. Tumor volume data were analyzed using two-way ANOVA, while tumor weight comparisons were performed using Student’s t-test. Vector-transfected cells were used as the control group. **P < 0.01, ****P < 0.0001.

The tumor-inhibitory effects of *RBM3* were further evaluated using a subcutaneous xenograft mouse model. Overall, nude mice injected with RBM3-overexpressing AGS cells showed significantly smaller tumors when they were compared to vector control group (**Figures 5A–C**). Histological analysis of the excised tumors revealed reduction in cellular proliferation. This was demonstrated by Ki67 immunohistochemistry, and increased apoptosis. Furthermore it was confirmed by TUNEL staining, in tumors overexpressing RBM3 (**Figure 5D**). Collectively, these results support a tumor-inhibitory role for RBM3 in gastric cancer. This inhibitory role is potentially adopted through the suppression of tumor growth and the promotion of apoptosis.

## 4 Discussion

Gastric cancer is a genetically heterogeneous malignancy. The inherited variations in GC interacts with tumor-intrinsic molecular programs to shape susceptibility and progression of disease. Our findings establish RBM3 as a bonafide tumor suppressor in gastric cancer (GC). These results were supported by convergent genetic and experimental evidence. The study demonstrated that germline variants linked to *RBM3* are associated with reduced risk of GC. Also, RBM3 expression is markedly downregulated in GC tissues and cell lines as compared to normal controls. Functionally, restoration of RBM3 in GC cells curbed malignant behaviors (reduction in proliferation, migration, invasion, and colony formation), whereas RBM3 knockdown had the converse effect. *In vivo*, RBM3 overexpression significantly suppressed xenograft tumor growth. This was complemented by lower levels of proliferation of Ki-67 and increase in apoptosis. These results strongly shows RBM3 as growth-inhibitory, pro-apoptotic factor in gastric cancer. These results also align with literature and suggest protective roles of RBM3 in cancer.

Our experimental findings are in strong agreement with previous gastric cancer–specific studies demonstrating that decreased RBM3 expression is significantly associated with advanced tumor stage, lymph node metastasis, distant metastasis, and aggressive clinicopathological features. Our results not only corroborate previous clinical observations linking RBM3 downregulation to aggressive gastric cancer phenotypes but also extend these findings by providing functional evidence that RBM3 directly suppresses gastric cancer cell proliferation, migration, invasion, and tumor growth. In esophageal cancer, benign epithelium shows uniformly high RBM3. Whereas, considerable subset of tumors particularly in advanced-stage & metastatic cases, displays reduction in RBM3 (18). Importantly, analogous associations between RBM3 downregulation and advanced tumor stage as well as metastatic progression have also been reported in gastric cancer, reinforcing the relevance of RBM3 loss to gastric tumor aggressiveness. This parallels our finding that GC tissues have lower RBM3 levels than that of non-tumor tissues. Although qPCR analysis demonstrated an overall reduction of RBM3 mRNA expression in gastric cancer tissues compared with adjacent normal tissues, some variability among individual paired samples was observed. Specifically, a subset of adjacent non-tumorous tissues exhibited RBM3 expression levels lower than the mean expression observed in tumor tissues. This variability likely reflects biological heterogeneity among patient samples as well as differences between mRNA and protein expression levels due to post-transcriptional regulation and protein stability mechanisms. In addition, Western blot analysis was performed on seven representative paired tissue samples rather than the entire cohort, which may not fully capture the variability observed in the larger qPCR dataset. Nevertheless, both transcript-level and protein-level analyses consistently supported an overall trend of reduced RBM3 expression in gastric cancer tissues. Conversely, patients having tumor with high RBM3 expression have generally better outcomes (19). A study of intestinal-type GC reported that RBM3-positive tumors were associated with significantly extended and prolonged survival (20). Similarly, a PRISMA-compliant meta-analysis by Gao et al. demonstrated that elevated RBM3 expression is significantly associated with favorable overall survival and improved clinicopathological characteristics across multiple tumor types, further supporting its prognostic value (21). Within gastrointestinal malignancies, colorectal cancer echoes this trend. The high tumor RBM3 has been correlated with improved survival of patient and enhanced chemotherapy responsiveness. These clinical links highlights broadly protective role for RBM3 in GI cancers which is in agreement with our experimental study in GC (22).

It should be noted that publicly available survival analyses from GEPIA2 and The Human Protein Atlas do not consistently demonstrate a statistically significant association between RBM3 expression and overall survival in gastric cancer patients. This discrepancy may be attributable to differences in cohort composition, sample size, tumor subtype distribution, technical platforms (RNA-seq versus qPCR or immunohistochemistry), and cutoff strategies used for dichotomizing RBM3 expression. In addition, bulk transcriptomic data may be confounded by tumor purity and stromal cell admixture, potentially obscuring tumor cell–specific associations. Therefore, while our tissue-based analyses indicate that reduced RBM3 expression is associated with more aggressive clinicopathological features, the prognostic value of RBM3 for survival outcomes in gastric cancer warrants further validation in larger, well-annotated clinical cohorts.

Despite this obvious protective pattern, role of RBM3 in cancer is not uniform across tissues. This highlights the importance of biological context. While our results and the prognostic data above support a tumor-suppressive function in GC, some studies have described pro-tumorigenic effects of RBM3 in different models. For instance, RBM3 has been shown to enhance the survival and proliferation of colon carcinoma cells under stress by post-transcriptionally upregulating COX-2, IL-8, and VEGF (23). In hepatocellular carcinoma models, RBM3 overexpression likewise promoted tumor cell growth *in vitro* and *in vivo*, partly via increasing a pro-oncogenic circular RNA. Such findings initially painted RBM3 as a potential oncogene in those settings (24). Merging these seemingly contradictory roles, recent reviews conclude that RBM3 can function as a double-edged sword in malignancy, behaving as a tumor promoter in some tissues (e.g. breast and colorectal cancer) but as a tumor suppressor in others. This dualistic behavior may reflect differences in tissue-specific co-factors or stimuli. In GC, RBM3’s net effect appears to be growth-inhibitory, whereas in colonic and liver tumor models under stress conditions, RBM3’s pro-survival, “cold-shock” attributes might favor tumor cell fitness (13). Our study firmly places GC on the side of RBM3 as a beneficial, suppressive factor. Furthermore, our study also helps to clarify this nuance by provision of direct mechanistic evidence in gastric cells.

Importantly, our work extends prior clinical observations by moving beyond correlation to causation. Prior to this study, RBM3 had been noted mainly as a prognostic marker in GC rather than a functionally validated tumor suppressor. Ye et al. (2017) observed via immunohistochemistry that RBM3 is frequently expressed in GC tissue. The patients with high tumoral RBM3 had significantly longer overall survival. Our results not only corroborate previous clinical observations linking RBM3 downregulation to aggressive gastric cancer phenotypes but also extend these findings by providing functional evidence that RBM3 directly suppresses gastric cancer cell proliferation, migration, invasion, and tumor growth (17). The forced RBM3 expression blunts GC aggressiveness, whereas its loss accelerates it. This cause and effect validation strengthens the argument that RBM3 is not merely correlative biomarker. But, it is also an active restrainer of progression of gastric tumor (25). Interestingly, other RNA-binding motif family members show similar patterns in GC. RBM4 is significantly downregulated in gastric tumors. This low RBM4 correlates with poor differentiation, nodal metastasis, and advanced stage (26). Patients with RBM4-low gastric tumors have significantly worse survival than those with RBM4 high tumors. The counterparts between RBM4 and RBM3 suggest that robust expression of certain RBM proteins is a symbol of healthier biology of gastric epithelium. The loss of these RNA regulators may unleash pro-tumorigenic programs (27). Our findings thus fit into broader narrative wherein cold-shock RBM3 act as guardians against malignancy in gastrointestinal tract, and their reduction marks a tipping point toward cancerous growth.

From a mechanistic point of view, the molecular functions of RBM3 provide rational foundation for its tumor-suppressive activity. RBM3 is a stress-inducible, glycine-rich RNA-binding protein. It regulates gene expression at post-transcriptional levels. It binds RNA, DNA and contributes to genomic integrity. It also modulates RNA splicing, stability, transport, and microRNA biogenesis (28). Through these functions, RBM3 acts as post-transcriptional regulator capable to reshape gene networks that control fate of cell. Its biological outcome appears context-dependent and driven by its downstream targets (29). In gastric cancer (GC), RBM3 displays a growth-inhibitory, pro-apoptotic role. RBM3 expression has been positively correlated with the pro-apoptotic protein Bax. It suggest that RBM3 may favor apoptotic signaling (30). Consistently, RBM3-overexpressing GC xenografts exhibit increase in apoptosis and reduction of proliferation. This indicates a shift from cell-cycle progression toward cell death. In addition, functional studies in gastric cancer models have demonstrated that RBM3 modulation influences tumor cell proliferation and apoptosis, further supporting its biological relevance in gastric tumor progression (31). RBM3 may stabilize transcripts which encodes tumor suppressors or cell-cycle inhibitors, while its loss may permit oncogenic transcript accumulation (32). RBM3 may also suppress tumor growth through stress-response and DNA damage pathways. It supports DNA replication checkpoints and genomic stability. It enables appropriate arrest of cell-cycle or apoptosis under genotoxic stress (33). Clinically, high RBM3 expression correlates with improved outcomes in chemotherapy-treated cancers. It suggest enhanced cell death induced by therapy. Identification of RBM3’s direct RNA targets in GC via transcriptome-wide approaches will be critical to fully define its tumor-suppressive network (34). In the present study, several RBM3-associated germline variants showed suggestive expression quantitative trait locus (eQTL) effects in stomach tissue. It indicated that inherited variation at the RBM3 locus may influence RBM3 transcriptional regulation. Given that RBM3 is an RNA-binding protein involved in RNA splicing, mRNA stability, and microRNA processing, these variants may affect gastric cancer susceptibility by modulation of RBM3 expression levels or alteration of post-transcriptional regulatory networks (22, 27, 28). Although we did not directly interrogate the functional consequences of individual RBM3 variants, recent studies have shown that RBM3 deficiency leads to transcriptome-wide splicing alterations and widespread RNA-processing dysregulation. It is therefore plausible that protective variants at the RBM3 locus contribute to gastric cancer risk reduction by preserving RBM3 expression or functional integrity in gastric epithelial cells. Further functional validation using allele-specific expression analysis and genome editing approaches will be required to establish causal mechanisms linking RBM3 genetic variation to gastric carcinogenesis. Importantly, the observed RBM3-associated phenotypes were consistently supported by multiple independent experimental approaches, including transcriptional validation by qPCR, functional assays evaluating proliferation, colony formation, migration, and invasion, as well as *in vivo* xenograft tumor growth analysis. The convergence of these independent lines of evidence strengthens the overall biological interpretation of RBM3 as a tumor-suppressive factor in gastric cancer.

Although an untreated control group is ideally included in *in vivo* tumorigenesis studies, vector-transfected cells were used as the experimental control in the present study. We acknowledge this as a limitation; however, repeating the *in vivo* experiments to include additional control groups would pose ethical and logistical challenges. Importantly, the observed tumor-suppressive effects of RBM3 overexpression were consistently supported by *in vitro* assays and multiple *in vivo* endpoints, thereby strengthening the overall conclusions. RBM3 modulation efficiency in this study was validated at both the transcript and protein levels. In addition to qPCR analysis, Western blot experiments confirmed successful RBM3 overexpression in AGS cells and effective RBM3 knockdown in HGC-27 cells. These results provide additional evidence supporting the reliability of the RBM3 modulation strategy used in our functional assays. However, two independent RBM3-targeting shRNA constructs were used and produced consistent knockdown efficiency and concordant functional phenotypes across multiple assays, including proliferation, colony formation, migration, invasion, and *in vivo* xenograft tumor growth. These results provide robust evidence supporting the functional impact of RBM3 modulation in gastric cancer models. Future studies incorporating quantitative protein-level analyses and mechanistic investigations will further strengthen the interpretation of RBM3-mediated signaling pathways. In addition, wild-type (non-transfected) cells were not included as an additional control group in the CCK-8 proliferation assays. However, non-targeting shRNA (sh-NC) and empty vector-transfected cells were used as standard experimental controls, which account for nonspecific effects associated with transfection procedures and serve as appropriate baseline references for evaluating RBM3-specific effects. These controls are widely accepted and routinely used in functional studies. Future studies incorporating wild-type controls may further strengthen validation of RBM3-mediated effects on gastric cancer cell proliferation.

Finally, these perceptions naturally point toward important future directions and translational opportunities. Further work is required to fully define its clinical and biological relevance. Future genetic studies should prioritize fine-mapping of RBM3-associated locus and functional validation of candidate variants. This should be done through allele-specific expression analyses and genome editing. This is done to establish direct causal mechanisms which link germline variation to RBM3 regulation and GC risk. The expansion of clinical validation is another key direction. Larger, multi-center cohorts which encompasses diverse populations and GC subtypes are needed to confirm prognostic value of RBM3 and to assess associations with treatment response, recurrence, and survival. Such studies would help determine whether RBM3 can serve as robust biomarker in clinical practice. Mechanistically, transcriptome-wide and proteomic analyses will be essential for the identification of RBM3-regulated pathways in GC. More physiologically relevant models such as gastric organoids and genetically engineered mice, will further clarify RBM3’s role within the tumor microenvironment and during early carcinogenesis. Finally, these findings open translational avenues. RBM3 expression could inform risk stratification or therapeutic decisions. Also, the pathways controlled by RBM3 may represent druggable vulnerabilities. Integration of genetics with functional studies offers a hopeful framework for personalized prevention of cancer and intervention in GC. Future work will include protein-level validation of RBM3 modulation and systematic characterization of downstream RBM3-regulated signaling pathways in gastric cancer models.

## 5 Conclusion

In summary, this study demonstrates that RBM3 plays a tumor-suppressive role in gastric cancer by the integration of genetic and functional evidence. Germline variants associated with RBM3 show consistent protective association with gastric cancer risk. RBM3 expression is significantly reduced in tumor tissues and correlates with aggressive clinicopathological features. Functional assays confirm that RBM3 inhibits gastric cancer cell proliferation, migration, invasion, and tumor growth. It also promotes apoptosis both *in vitro* and *in vivo*. Collectively, these findings identify RBM3 as a genetically informed tumor suppressor. It also highlight its potential value as biomarker and therapeutic target in gastric cancer.

## Data Availability

The original contributions presented in the study are included in the article/**Supplementary Material**. Further inquiries can be directed to the corresponding author.
