# Supplementary material for: RBM3-associated germline variants and their functional role in gastric cancer susceptibility and progression
Source: Front Oncol. 2026 Apr 1;16:1790197. doi: 10.3389/fonc.2026.1790197 (PMC13079030; doi:10.3389/fonc.2026.1790197)
Supplement: Supplementary file 1 [file Table1.docx]

Table 1. Information about exposure factors and outcome factors

| Type | Trait | ID | Population | Sample_Size | nSNP |
| --- | --- | --- | --- | --- | --- |
| exposure | RNA-binding protein 3 levels (RBM3.12747.89.3) | GCST90242696 | EUR | 3301 | 8511609 |
| outcome | Cancer of stomach (PheCode 151) | GCST90041806 | EUR | 456348 | 11796992 |
