# Supplementary material for: RBM3-associated germline variants and their functional role in gastric cancer susceptibility and progression
Source: Front Oncol. 2026 Apr 1;16:1790197. doi: 10.3389/fonc.2026.1790197 (PMC13079030; doi:10.3389/fonc.2026.1790197)
Supplement: Supplementary file 4 [file Table4.docx]

Supplementary Table 4. Co-positioning analysis

| SNP | PP.H4.abf |
| --- | --- |
| rs138485358 | 0.003166962 |
| rs41272307 | 0.00252378 |
