# Supplementary material for: RBM3-associated germline variants and their functional role in gastric cancer susceptibility and progression
Source: Front Oncol. 2026 Apr 1;16:1790197. doi: 10.3389/fonc.2026.1790197 (PMC13079030; doi:10.3389/fonc.2026.1790197)
Supplement: Supplementary file 5 [file Table5.docx]

| **Supplementary Table 5. Primary antibodies used in this study. The table provides information on antigen targets, manufacturers, catalog numbers, and working dilutions for Western blot (WB) and immunohistochemistry (IHC) analyses.** | | |
| --- | --- | --- |
| Antigens | Manufacturers | Application |
| Anti-RBM3 antibody | ab134946, ABCAM, Cambridge, UK | 1:1000 for WB 1:300 for IHC |
| Ki-67 | #AF0198, Affinity Biosciences, Cincinnati, OH, USA | 1:200 for IHC |
| β-actin | #T0023, Affinity Biosciences, Cincinnati, OH, USA | 1:5000 for WB |
| WB, Western Blot; IHC, Immunohistochemistry. | | |
